# Supplementary material for: Are carbon emissions trading and green financial instruments synergistic? -Comprehensive quantitative research based on content analysis
Source: PLoS One. 2024 Mar 7;19(3):e0298601. doi: 10.1371/journal.pone.0298601 (PMC10919601; doi:10.1371/journal.pone.0298601)
Supplement: S1 Data — (ZIP) [file pone.0298601.s001.zip › Data/地级市Z_F工作报告.zip!/╔╜╢1⁄2/╢1⁄2╙¬╩╨/└┤╫╘╣1⁄2╓┌║┼í╛┬φ┐╦╩2╛▌═°í┐_2017.docx]

各位代表：

现在，我代表市人民政府向大会报告工作，请予审议，并请市政协委员和其他列席会议的同志提出意见。

一、过去五年工作回顾

市第七届人民政府就职以来，在以习近平同志为核心的党中央和国务院、省委、省政府、市委的坚强领导下，全市上下全面落实党的十八大和十八届三中、四中、五中、六中全会精神，深入学习贯彻习近平总书记系列重要讲话精神，全力实施黄蓝国家战略，统筹推进稳增长、促改革、调结构、惠民生、防风险各项工作，经济社会发展取得显著成绩。

五年来，我们克服重重困难，坚定不移推进转型发展，经济平稳较快增长，综合实力迈上一个大台阶。2016年，全市实现生产总值3479.6亿元，是2011年的1.6倍，年均增长9.4%。规模以上工业主营业务收入达到1.3万亿元，年均增长10%，过百亿企业32家，过500亿企业2家，23家企业入围山东省民营企业百强。一般公共预算收入达到221.9亿元，年均增长10.7%。金融机构各项存款余额3989.7亿元、贷款余额3507.5亿元，分别是2011年底的2.1倍和2.4倍。

产业结构明显优化。三次产业结构由3.7:71.6:24.7调整为3.5:62.2:34.3。加快新型工业化，石油化工及盐化工、橡胶轮胎及汽车配件、有色金属成为千亿级产业，工业主导产业80%以上的技术装备达到国内先进水平，高新技术产业比重提高4.3个百分点。“三去一降一补”全面实施，在全省率先开展化工产业“三评级一评价”，淘汰一批落后产能，产业质量层次明显提升。服务业增加值增长1.8倍。强化创新驱动，研发经费占生产总值比重达到2.62%，中国和山东名牌产品达到145个，被评为全国质量魅力城市、国家首批知识产权示范城市，连续六次荣获全国科技进步先进市称号。

农业现代化水平显著提高。被整建制列为国家现代农业示范区，园区化率居全省首位。优势主导产业健康发展，万头奶牛场达到5个，水产养殖面积达到197万亩，工厂化食用菌生产能力达到40万吨。农业龙头企业发展到650家，家庭农场发展到1039家，农民专业合作社实现涉农行政村全覆盖。大规模开展农田水利建设，农业生产条件明显改善。新型农民学校年均培训40.2万人次。

改革开放步伐加快。38项国家和省级改革试点顺利推进。商事制度、财税、金融、农村、企业、公立医院、教育、事业单位等改革取得新突破。累计完成进出口4170.3亿元，位居全国外贸百强市第44位；实际利用外资10.2亿美元，引进8家世界500强企业。马德里国际注册商标总量居全省第2位。橡胶轮胎展、石油装备展越办越好，首届有色金属展取得良好效果。对口支援任务圆满完成。

区域融合发展取得新成效。胜利油田克服严峻挑战，全力提质增效。完善油地协商对接机制，实施合作事项56个。济军综合训练基地改革发展迈出新步伐。油地校三方共建胜利学院开创新局面。广饶、垦利进入全国工业百强县，东营区进入全国综合实力百强区，河口、利津发展取得新突破。

五年来，我们抢抓历史机遇，坚定不移抓谋划抓推进抓落实，事关长远的大事要事实现突破，发展后劲更加充足。成为京津冀协同发展城市、环渤海地区重要节点城市。东营综合保税区封关运营。黄河三角洲农业高新区正式成立。东营作为沿海高铁和京沪高铁二通道重要节点城市列入国家规划。德大铁路、济东高速公路建成通车。东营港新增码头38个，防波堤工程获得交通运输部和省资金支持，10万吨级航道工程列入交通运输部“十三五”规划。广利港建设快速推进，港区和防波堤建设全面展开。长深高速西延到济南机场列入省高速公路网规划。胜利机场增开优化航线7条，中国商飞公司试飞基地建设取得积极进展。7家企业获得使用进口原油资质和允许量2000万吨。黄河南展区改造建设获得国家支持。垦利撤县设区顺利完成。东营获得国家公共文化服务体系示范区创建资格，成功创建为全国文明城市。

五年来，我们坚持生态宜居定位，坚定不移改善生态环境，城市功能日益完善，城乡面貌发生巨大变化。中心城“大空间、大绿地、大水面、大湿地”特色初步形成，建成区面积达到188.2平方公里，荣获中国人居环境奖。大剧院、科技馆、规划展览馆等建成投用，建设改造东一路、西二路等城市道路190公里。新建城市公园15个、街头游园24个、城市绿道107.7公里。中心城建设管理体制进一步理顺，拆违治乱工作走在全省前列。统筹城乡发展，全市改造城中村52个、老旧小区446个、棚户区48个，开工建设农村新型社区62个。持续推进农村“改路、改电、改校、改房、改水、改厕、改暖”，实施乡村文明建设专项行动，城乡一体化的环卫运行机制实现全覆盖。

生态建设成效明显。实施了金湖银河、森林湿地公园、广利河生态廊道等重大生态工程。持续开展大规模植树造林，启动实施三年增绿计划，五年新增造林31.4万亩，创建为国家园林城市。自然保护区恢复改造湿地32万亩，综合执法获省政府批复。持续开展水气污染整治，实施环保重点工程320项，新建改造城乡污水处理厂13座、垃圾处理场1个，治理淘汰黄标车4.1万辆，主要河流基本消除劣五类水体，环境优良天数明显增加。

五年来，我们顺应群众期盼，坚定不移保障改善民生，社会更加和谐，人民群众幸福指数不断提升。持续加大民生投入，每年实施一批民生实事。城乡居民收入分别达到41580元、14999元，年均增长9.2%、11.1%。新增城镇就业23.5万人，城镇登记失业率控制在2%左右。在全省率先整合城乡居民基本医疗保险、基本养老保险制度，建立城乡居民大病保险、临时救助等制度，各项社会保障标准居全省前列。省定标准贫困人口全部脱贫。

社会事业繁荣发展。各级各类教育协调发展，县区义务教育均衡发展整体通过国家验收，市一中、二中、特校新校区投入使用，加快解决城镇中小学大班额问题，教育教学质量稳步提升。医疗卫生事业健康发展，市人民医院创建为三甲医院。文化惠民工程深入实施。黄河口国际马拉松赛成为国际田联金标赛事。连续四届成为全国双拥模范城市。人口计生、妇女儿童、慈善、残疾人、养老、老龄事业健康发展，统计、档案、气象、防震减灾、人民防空、民族宗教、外事侨务、对台工作取得新的成绩。

社会治理不断加强。社区规范化建设深入推进，村居“两委”换届全面完成。“六五”普法圆满完成。一批信访积案得到有效解决。社会治安力度加大，城市公共安全专项行动扎实有效，食品药品安全监管全面加强，应急管理水平不断提升，社会秩序持续稳定。

我们高度重视政府自身建设，严格落实中央八项规定精神，深入开展党的群众路线教育实践活动、“三严三实”专题教育、“两学一做”学习教育，大力整治“四风”突出问题。权力、责任、审批“三张清单”全面推行，行政复议和行政应诉工作走在全省前列。认真执行市人大及其常委会的决定决议，自觉接受各方面监督。严格落实党风廉政建设责任制，一批违纪违法行为得到严惩。

2016年，宏观经济下行压力加大，特别是油价低迷、欧美“双反”对我市经济造成了雪上加霜的严峻困难。我们密切关注形势变化，及时出台措施，推动经济缓中趋稳、稳中向好，全市生产总值增长7%；一般公共预算收入同口径增长2.4%，其中市级增长10%；规模以上工业增加值增长6.2%，21家企业入围山东省企业百强、居全省首位；实现进出口995.9亿元，增长24.7%，增速居全省第2位。虽然有些指标增幅较低，但总体好于同类资源型城市。尤其是一些先导性经济指标较好，工业用电量增长10.1%；金融机构各项贷款余额比年初增加476亿元，增速居全省第2位，不良贷款率处于全省较低水平。

各位代表！过去的五年是我们经受住各种困难考验，取得巨大成就的五年，也是我们在加快建设美丽幸福新东营道路上奋勇前进的五年。五年的不懈奋斗，奠定了率先全面建成小康社会、创造更加美好生活的坚实基础，积累了应对复杂环境、战胜风险挑战的宝贵经验，开启了践行新理念、推动新发展的重要阶段。这些成绩的取得，是在中央、省委、省政府和市委正确领导下，全市上下、油地军校、社会各界团结奋斗、携手共建的结果。在此，我代表市政府，向付出辛勤劳动的广大干部群众，向积极支持政府工作的人大代表、政协委员和社会各界人士，表示崇高的敬意和衷心的感谢!

在看到成绩的同时，我们也清醒地认识到，我市经济社会发展还存在不少问题，政府工作还有很多不足。原油价格仍在低位徘徊，企业生产经营面临诸多困难。供给侧结构性改革推进不快，自主创新能力不强，产业转型升级任重道远。经济发展质量效益不高，财政增收压力很大。安全生产存在薄弱环节，水气污染治理任务依然艰巨。重大基础设施仍不完善，公共服务均等化水平有待提高。政府效能与群众期盼还有差距，有的工作人员缺乏敢于担当、勇于进取的精神。对此，我们将坚持问题导向，采取针对性措施，认真加以解决。

二、今后五年的总体要求和原则方向

今后五年，是东营资源城市转型攻坚期、战略地位提升跨越期、全面建成小康社会决胜期。市第六次党代会，明确了今后五年的总体思路和奋斗目标，为东营的发展指明了方向。我们要坚定信心，敢于担当，撸起袖子加油干，扑下身子抓落实，奋力开创东营事业发展的新局面。

根据市第六次党代会部署，今后五年政府工作的总体要求是：高举中国特色社会主义伟大旗帜，深入学习贯彻习近平总书记系列重要讲话精神，紧紧围绕协调推进“四个全面”战略布局，自觉践行创新、协调、绿色、开放、共享发展理念，按照建设黄河三角洲中心城市、我国重要的石油基地的定位，持续实施黄蓝国家战略，以提高发展质量效益为中心，以推进供给侧结构性改革为主线，加快形成引领经济发展新常态的体制机制和发展方式，统筹推进经济建设、政治建设、文化建设、社会建设、生态文明建设和党的建设，率先全面建成小康社会，加快建设繁荣富裕、生态秀美、文明和谐、宜居宜业的美丽幸福新东营。

今后五年，贯彻总体要求，实现奋斗目标，必须把握好原则方向，在四个方面持续发力。

——在产业转型升级上持续发力，着力增创经济发展新优势。把工业转型升级作为重中之重，深入实施“中国制造2025”东营方案，加快推进新型工业化，使四大产业集群规模档次和质量效益明显提升，成为全国领先、世界有影响力的先进制造业基地，一批新兴产业成为重要支柱产业。加快发展现代农业，率先实现农业现代化。重点突破服务业，打造区域性服务中心。着力培育新增长点，东营港石化产业基地、黄河三角洲农业高新区、东营综合保税区、空港产业区、广利港临港产业区、东营高新区发展实现重大突破，成为引领产业发展的强大引擎。大力强化创新驱动，科技创新体系日益完善，创新创业蓬勃发展，科技进步对经济增长的贡献率大幅提高，建设区域性创新中心。

——在生态文明建设上持续发力，着力增创绿色发展新优势。牢固树立“绿水青山就是金山银山”的理念，铁腕治理水气污染，大幅提高林木绿化率、四类以上地表水比例和环境优良天数，加强自然保护区生态保护和修复，基本形成绿色发展产业结构、生产方式、生活方式，积极创建国家生态文明示范区。突出“大空间、大绿地、大水面、大湿地”特色，建设生态城镇、美丽乡村，创建成为国家生态园林城市、联合国人居奖，彰显湿地之城、生态之城的魅力，让生态宜居成为东营闪亮的城市名片。

——在重大基础设施建设上持续发力，着力增创开放发展新优势。把重大交通设施建设摆上战略位置，构建起“六纵、八横、一环”的骨干公路网，实现东营人民的高铁梦，东营港建成亿吨大港，广利港吞吐能力达到2000万吨，胜利机场成为区域性中心空港、国家重要的民机试飞基地，成为横贯东西、纵贯南北的区域性交通枢纽城市。坚持开放发展理念，深度融入国家“一带一路”和重大区域发展战略，加快引进来、走出去步伐，打造沿海开放新高地。全面深化各项改革，使东营的发展更加活力迸发。

——在改善民生上持续发力，着力增创和谐发展新优势。践行以人民为中心的发展思想，以更高标准、更大力度保障改善民生，让群众有更多获得感、更高满意度。实现更加充分的就业，持续增加居民收入。完善社会保障体系，保障救助标准与经济发展同步增长，率先完成脱贫攻坚任务，人人享有基本生活保障。进一步提升公共服务水平，让群众不再为看病、上学犯难，所有老年人都能安享晚年，住房困难家庭实现住有所居，家家用上天然气。创建成为国家公共文化服务体系示范区，群众精神生活更加丰富。深入推进平安东营建设，社会秩序更加和谐稳定。

三、2017年政府工作总体安排

2017年，党的十九大将胜利召开，做好今年的工作意义重大。主要预期目标是：生产总值增长7%左右，一般公共预算收入增长6%左右，城镇新增就业3.3万人，城镇登记失业率控制在3.5%以内，进出口适度增长，居民消费价格涨幅控制在3%左右。全面完成省下达的年度节能减排等约束性指标。重点做好以下十个方面的工作：

（一）积极扩大有效投资

坚定不移实施项目带动战略，突出抓好105个市级重点项目、204个市政府投资项目、114个水气污染治理项目、11个民生实事项目，这些项目总投资1365亿元，今年计划投资528亿元。用好政策性资金，论证一批优质项目，加大政府债券和上级预算内资金、专项建设基金、政策性贷款争取力度。着力抓好政府投资项目管理，严格落实招投标、政府采购、工程监理等制度规定，加强工程审计，确保项目质量。把招商引资作为扩大投资、推动发展的重大引擎，坚持引资与引智并举，完善工作推进机制，制定优惠政策，掀起新一轮招商热潮，努力引进一批世界500强、央企和行业领军企业。积极扩大民间投资，加快实施一批PPP项目。优化项目服务，对重大项目实行全程代办、联审联批、重点督办。

（二）着力推动工业内涵式发展

重点抓好四大产业集群。石油化工及盐化工产业，以产业集聚、延长链条为主攻方向，认真落实“1+N”布局规划，争取东营港国家级石化产业基地尽快批复，加快乙烯、芳烃等重大项目立项，大力培育高端化工产业体系；扎实开展“四评级一评价”，倒逼落后产能退出。橡胶轮胎及汽车零部件产业，坚持走高端化、差异化、品牌化的路子，加快发展轮胎、汽车零部件高端产品，鼓励发展特种车、专用车整车制造。有色金属产业，依托龙头企业，引进战略合作伙伴，提高环保冶炼和精深加工水平，打造国内一流的铜产业基地。石油装备产业，加强与国际石油装备巨头的合资合作，加快开发成套设备和高新产品，推广“生产+服务”的营销模式，扩大国际市场占有率。围绕四大产业集群，建设一批高水平的公共创新平台、检测平台、产品展销平台和物流平台，力争今年四大产业集群产值突破万亿元。

改造提升传统产业，加快发展新兴产业。启动新一轮高水平技术改造，支持造纸、纺织、机械制造等产业加快改造提升，以市场需求为导向，开发更多适销对路的新产品。选择有基础有条件的领域集中突破，培育壮大新能源、新材料、新医药、高端装备制造等产业，今年实施好总投资174亿元的27个重点项目，努力打造一批百亿级新兴产业。

大力提高企业竞争力。引导广大企业弘扬专注精神、工匠精神、创新精神，加强技术创新、管理创新和产品创新，打造更多的“百年老店”。支持大企业借助国际一流高校院所的力量，以行业顶尖企业为标杆开展自主创新，积极创建制造业创新中心；引导中小企业深化产学研结合，朝着“专、精、特、新”方向发展。支持具备条件的企业开展市场化、法治化债转股。加快企业规范化公司制改制，建立现代企业制度，今年完成改制100家。鼓励企业扩大直接融资，力争10家企业上市挂牌。认真落实领导干部联系企业和驻企联络员制度，帮助企业解决实际困难。支持企业兼并重组，积极开展对外合资合作，进一步做大做强。

深入实施质量强市和品牌战略。逐行业制定质量提升计划，支持企业主导或参与制订行业标准、国家标准和国际标准，提升研发设计能力。推动马德里国际商标注册，支持企业开展品牌并购，创建一批知名自主品牌。加强知识产权保护，争创国家知识产权强市。通过企业、政府、社会一起努力，把华泰、蓝海、科瑞、方圆、万达、利华益等知名企业形象树得更好，让黄河口大闸蟹、黄河口大米等黄河口地理标志农产品卖得更好，把橡胶轮胎、石油装备生产基地等区域品牌叫得更响。

深入实施人才强市战略。加快推进“黄河三角洲学者”、技能振兴专项行动等重大人才工程，加强与国家“千人计划”专家及团队的合作，支持企业面向全球引进行业领军人才，引进高水平的创新创业团队。企业家是最重要的人才。要深入实施企业家培训三年计划，大规模组织企业家走出去，瞄准国内外一流企业开展对标学习。支持企业家干事创业，形成全社会尊重创业、尊重企业家的浓厚氛围。

（三）加快发展现代农业、建设美丽乡村

做大做强特色优势产业。深入推进农业供给侧结构性改革，增加绿色优质农产品供给。畜牧业，发挥龙头企业带动作用，抓好高端乳制品加工等重点项目建设，打造优质畜产品生产基地。渔业，加快实施工厂化对虾养殖等重点项目，建设50万亩海洋生态牧场，进一步提升大闸蟹等特色产品品质。食用菌产业，引进研发高档珍稀食用菌品种，搞好产品精深加工。种植业，扩大高效作物种植面积，推广“粮经饲”三元种植模式，巩固提升粮食生产能力。

加快发展工厂化农业。坚持以工业理念发展农业，引导工商资本发展设施农业。加大对农业龙头企业的扶持力度，新上一批农产品精深加工项目，建设一批智能化种植养殖工厂。重点扶持30个家庭农场、20个农民专业合作社。加快发展“互联网+农业”，培育一批农业电商平台。建设一批产品可查询、质量可追溯的标准化生产基地。支持市现代畜牧业示范区加快发展。

着力改善农业发展条件。实施“渤海粮仓”等一批科技示范工程，推广应用农业先进适用技术，大力发展节水农业和水肥一体化。实施南水北调配套和黄水东调应急工程，积极推进小清河复航。综合治理支脉河、太平河等河道，实施王庄、麻湾等灌区配套改造和田间水利工程。抓好农村承包土地经营权抵押贷款试点，开展农村集体产权制度改革试点，深化供销社综合改革。积极探索农村土地所有权、承包权和经营权“三权分置”，鼓励发展适度规模经营。

积极开展美丽乡村标准化建设。严格按照省级创建标准，全面落实各项工作任务。坚持不懈抓好农村“七改”工程，今年全部完成农村旱厕改造，建立完善长效运行机制；启动实施天然气下乡工程，今年为具备条件的783个村庄、社区接通天然气，实现城乡供气一体化；改造农村公路300公里、农村电网330公里、农村危房1100户。深化农村环境综合整治，巩固扩大乡村文明建设成果。

（四）推动服务业加快发展

大力发展金融业，加强政银企合作，支持银行机构强化产品和服务创新，满足企业多元化融资合理需求；高度重视风险防范工作，严厉打击恶意逃废银行债务行为，维护良好的金融生态环境；支持法人金融机构上市挂牌，加快发展；继续抓好新型农村合作金融试点。加快发展旅游业，以创建全域旅游示范市为抓手，加强黄河口湿地的保护和修复，抓好黄河口生态旅游区国家5A级景区创建；支持地热开发利用，发展温泉旅游；加快培育休闲农业、乡村度假等乡村旅游新业态。积极发展现代物流业，依托主导产业和重大交通设施，规划建设一批物流园区，培育一批大型物流企业。加快提升传统商业，推进实体商业创新转型，深化与阿里巴巴、京东等知名电商的合作。积极发展科教产业，规划建设高端教育园区。大力发展养老服务业，扩大购买居家养老服务覆盖面，加快新型养老社区建设。规范住房租赁市场，多渠道化解库存，促进房地产市场平稳健康发展。

（五）提升新型城镇化发展水平

牢牢把握生态宜居城市定位，以创建国家生态园林城市为抓手，全面提升规划建设管理水平。

完善提升城市规划。优化中心城空间布局，统筹推进东城、西城、垦利三个组团的规划建设。开展2020年—2030年城市规划编制工作，加快实施城市总体规划和土地利用规划“两规合一”，积极推进“多规融合”。今年实现中心城区控规和城市设计全覆盖。

抓好中心城重点项目建设。城市生态环境方面，加快建设24平方公里的森林湿地公园，高速公路以东的森林体验区，今年“十一”建成对外开放，高速公路以西的五湖景观区，争取明年“十一”对外开放；加快实施与之相配套的“一水库两管线”工程，争取今年5月底建成纯化水库、广北水库至耿井水厂的两条输水管线，明年底完成广北水库改造提升工程，替代耿井水源，提升供水水质。继续推进利三沟水系完善工程，今年“十一”建成，在东城核心区打造一条靓丽的带状公园。规划建设白鹭湿地公园，完善提升园博园，改造建设清风湖、锦华等城市公园。抓好东二路、东三路、北一路、西三路等道路绿化提升，新建街头游园17处、城市绿道88公里，全面推进拆墙透绿、拆墙建绿。今年中心城区新增绿化面积396万平方米。城市道路方面，加快南一路快速路（西一路至华山路段）建设，争取年底建成通车；统筹推进东二路、西一路、西二路、德州路、登州路、华山路等主次干道建设，打通城区“断头路”，建设完善40条城市支路，缓解交通拥堵问题。片区改造方面，启动实施东营河及北二路片区综合整治工程，实施东城商贸城、国际汽贸城等片区改造；今年全市全部完成1998年以前建成的老旧小区改造，力争明年全部完成棚户区和城中村改造。市政公用设施方面，改造完善中心城供水、供热、供气管网，实施东一路、府前大街、黄河路亮化工程，对部分单位庭院按照海绵城市标准进行改造，推进地下综合管廊建设。智慧城市建设方面，实施好政务云平台、政务信息资源共享交换平台等基础工程。对今年实施的各类城建项目，一定坚持标准，严格把关，确保效果，建成群众满意工程。

大力推行城市精细化管理。完善城市管理体制，全面提升标准化、网格化、智能化、机械化和市场化水平，用3至5年时间，实现市和县区城市精细化管理全覆盖、长效化。深入推进拆违治乱专项行动，年底前全面完成违法建设治理任务，该拆除的坚决拆除，该处罚的依法处罚到位，坚决杜绝新增违法建设。突出抓好公路铁路沿线环境综合治理，彻底拆除违建、清脏治乱，全面绿化提升，打造整洁有序、充满生机、欣欣向荣的生态长廊。

统筹推进县城和小城镇建设。支持各县区加强县城规划建设管理，推进产城融合，完善城市功能，建设各具特色的现代化小城市。培育一批新生小城市、特色小镇，建设一批示范街区和特色社区，继续抓好黄河南展区新型农村社区建设。加快推进人口市民化工作，今年常住和户籍人口城镇化率均提高2个百分点以上。

（六）加强重大基础设施建设

编制完善全市综合交通规划，着眼长远，统筹兼顾，科学规划布局铁路、轻轨、机场、港口、公路、车站、管道等各类设施，为将来有序建设预留空间，避免前后冲突、相互“打架”。编制以高速铁路站点为核心的综合枢纽规划，在中心城南部形成一个以高铁站为中心，各类交通线路相互连接、四通八达的客运枢纽，方便旅客快速换乘。编制管网规划，合理布局原油、成品油、天然气等管道设施，逐步替代危化品车辆运输，降低企业运输成本，减少车辆污染排放，改善道路交通环境。

把高铁建设作为大事来抓，积极向上争取，加快推进“天津—东营—潍坊”高铁和“滨州—东营—潍坊滨海”快速铁路前期工作，力争早日开工建设。加快建设黄大铁路、东营港疏港铁路，实施淄东铁路扩能改造，积极推进广利港疏港铁路前期工作。加快东营港10万吨级航道、25万吨级单点系泊、南防波堤建设。完成广利港防波堤延伸、南防波挡沙堤等工程，年内实现通航。规划建设红光新渔港。加快推进市域高压天然气管网和东营港至郝家原油管道工程。继续增开优化胜利机场航线航班，加快中国商飞试飞基地建设，争取跑道延长工程尽快投用。年内开工建设四条骨干道路，包括河口蓝色经济产业园至广饶李鹊的河广大道、庐山路向南延伸至广饶稻庄、南二路向西跨黄河延伸至滨州界、S507董集至利津黄河大桥段改建。实施长深高速广饶至高青段建设。加快推进大唐电厂、500千伏垦东输变电工程。

（七）持续改善生态环境

大规模开展国土绿化。深入实施三年增绿计划，重点抓好环城林、环镇林、围村林、绿色通道、农田林网等工程，今年造林17.3万亩，确保三年实现新增造林40万亩的目标。积极开展绿化示范乡镇、示范村、美丽校园和生态厂区创建活动。制定湿地保护修复计划，以自然保护区为重点，修复湿地41.3万亩，封育天然柽柳林8000亩。

深入实施水气土污染整治专项行动。全面推行河长制，突出抓好溢洪河、广利河等重点河流综合整治，加快推进雨污分流改造、污水处理厂提标、农村面源污染防治，实施六干排等生态修复工程，全市河流全面稳定达到地表水五类水质。对涉水污染源实行排放总量与浓度“双控制”，全面整治城乡黑臭水体。突出PM2.5和挥发性有机物的防治，完善大气自动监控系统，抓好21个重点区域异味治理，年内所有产生挥发性有机物的企业完成治理任务。实施“工业绿动力”计划，加强散煤治理，所有燃煤电厂和10吨以上燃煤锅炉全部完成超低排放改造，全部淘汰10吨以下燃煤锅炉。抓好城市面源污染防治，落实“抑尘、控车”等综合措施。严禁露天焚烧垃圾、秸秆。持续开展加油站清理整顿和油品质量专项检查。以耕地为重点，有序开展土壤污染治理与修复。扎实开展环境监管执法年活动，严厉打击偷排偷放行为，公开处理一批环境违法案件，让违法者付出代价。

加强资源节约综合利用。大力发展循环经济，开展垃圾分类试点，加强畜禽养殖废弃物资源化利用。强化危险废物规范化管理，规划建设全市危险废物综合处置场所。严格落实能源节约和资源保护制度。

（八）深入推进改革开放

深化经济领域重点改革。推进商事制度改革，全面推行“多证合一、一照一码”登记制度。深化财税体制改革，加强预算绩效管理，实行中期财政规划管理；大力压减“三公”经费等一般性支出，继续推进专项资金清理整合，加大税源管控和开源增收力度；逐步实现全市基本民生标准、农业转移人口市民化待遇、工资性收入政策、运转经费标准和公共设施建设标准“五个基本统一”。加快重点领域价格改革。深化国有企业改革，不断完善公司治理结构，对市属国有企业进行清理规范，加强国有资产管理。推进生态文明体制改革，落实生态红线制度，建立完善生态补偿机制。深化医药卫生体制改革，加快分级诊疗制度建设，抓好公立医院综合改革。继续推进教育、事业单位、编制人事、综合行政执法等改革。

提升开放型经济发展水平。加快优化出口产品结构，支持企业扩大先进技术、关键设备和核心零部件进口，加快推进科瑞跨境电商等平台建设，促进对外贸易平稳增长。继续抓好原油进口资质和允许量争取工作，加大原油进口力度。加强东营综合保税区运营管理。推进海关综合监管平台和电子口岸建设。支持优势企业开展国际产能合作，推动装备、技术、标准和服务“走出去”。高水平办好石油装备展、橡胶轮胎展、有色金属展等大型展会。积极承接北京非首都功能疏解和京津产业转移。

（九）统筹区域协调发展

支持胜利油田积极应对低油价，加快改革发展。深化油地合作共建，携手做好创新驱动、城市建设、深化改革、维护稳定四篇大文章，配合做好“四供一业”移交工作，共建幸福美好家园。深化与中国石油大学合作。打通胜利油田、石油大学人才技术和科研成果走向市场的通道。支持驻地部队改革建设，深化双拥共建，打造军民融合深度发展示范市。加快发展县域经济，促进强县争先进位，支持利津跨越赶超。

（十）着力保障改善民生

打赢脱贫攻坚战。年内实现市定标准贫困人口全面脱贫。做好脱贫攻坚兜底保障，实现农村低保标准与市扶贫标准“两线合一”，稳定实现农村贫困人口不愁吃、不愁穿，义务教育、基本医疗和住房安全有保障。统筹解决好城市贫困问题。

促进就业创业。做好结构调整、淘汰落后产能中失业人员的再就业，统筹农村转移劳动力、就业困难人员、退役军人等群体就业，抓好企业下岗失业人员技能培训和再就业工作。深入推进大众创业万众创新，组织开展小微企业创业素质能力培训，创建一批省级和市级创业孵化基地、示范园区。实施新一轮大学生创业引领计划，促进多渠道创业就业。完成农民工职业技能提升、权益保障和公共服务三年行动计划。推行工资集体协商制度，规范劳动用工管理。

强化社会保障。继续提高城乡低保、特困人员救助供养标准。实施全民参保计划，推动实现法定人员全覆盖。建立救急难工作机制，启用大救助信息平台。落实被征地农民参加基本养老保险政策，完善居民大病保险和职工长期护理保险制度，建立职工大病保险制度，探索建立居民长期护理保险制度。支持发展老龄、残疾人、慈善和社会福利事业。

加快发展教育。以教育现代化为总目标，提升各级各类教育发展水平。全面完成第二期学前教育三年行动计划，扩充公办幼儿园和普惠性民办幼儿园数量。促进义务教育优质均衡发展，优化普通高中教育资源配置，完成市一中、二中二期建设工程。年内投资29亿元，新建改造44所学校，解决大班额问题。大力发展职业教育，支持东营职业学院创建国家优质高职院校。鼓励民办教育发展。开展教育现代化学校、乡镇、县区三级创建活动。加快建设市青少年示范性综合实践基地。启动建设老年大学。

建设健康东营。完善基层医疗卫生服务体系，提升基层医疗卫生服务能力。强化公共卫生服务项目精细化管理，推动公共卫生服务均等化。加强妇幼服务和疾病防控能力建设。加快中医药事业发展。建设人口健康信息平台，全面提高出生人口素质。加强食品药品安全监管，积极创建省级食品安全城市。广泛开展全民健身活动，办好国际马拉松赛、公路自行车赛。

繁荣发展文化。加快国家公共文化服务体系示范区创建。完成黄河文化馆展陈和市美术馆改造，提升基层综合文化服务中心服务效能，推进乡村剧场和数字文化广场建设。加快市、县综合档案馆和档案信息化建设。深化群众性精神文明创建活动，巩固扩大文明城市创建成果。支持骨干文化企业加快发展，培育一批文化新业态。实施吕剧振兴工程，打造黄河口文化品牌。加强网络文化建设。

创新社会治理。深化平安东营建设，完善立体化、信息化社会治安防控体系，加强油区治安综合整治，强化警民联防联治。集中开展“两打一整治”专项行动，严厉打击黑恶霸痞犯罪，打击盗抢骗犯罪，整治社会治安乱点。完善社区治理机制和服务体系。加强和改进信访工作。认真开展“七五”普法，建立健全“一村一法律顾问”“一村一警务助理”制度。进一步加强人民调解、社区矫正和安置帮教工作，建立完善公共法律服务体系。深化道路交通“平安行·你我他”行动，大力普及交通规则和汽车文明，继续实施农村公路安全生命防护工程。加强社会信用体系建设，完善信用联合惩戒制度。

加强安全生产工作。狠抓安全生产责任落实，建立完善风险分级管控和隐患排查治理双重预防机制。加大执法检查力度，深化危化品、道路交通、消防安全、建筑施工、特种设备等领域专项整治和打非治违，全面提高事故防范能力，杜绝重特大生产安全事故发生。

办好民生实事。（1）实施农村天然气村村通。（2）改造敬老院设施，提升养老服务水平。（3）实施农村旱厕改造工程。（4）实施老旧住宅小区改造工程。（5）实施街头游园及绿道建设工程。（6）推进畜禽养殖废弃物无害化资源化处理。（7）缓解适龄幼儿入园难，解决中小学大班额问题。（8）实施便民服务一卡通工程。（9）采取综合措施防治蚊虫。（10）提升居民小区安全防范水平。（11）建设全民健身中心。

四、加强政府自身建设

一要全面从严治政。牢固树立“四个意识”，坚决维护以习近平同志为核心的党中央权威。持续推进“两学一做”学习教育常态化制度化。严格落实中央八项规定精神，持之以恒反对“四风”。坚持把纪律规矩挺在前面，健全完善从严治政的制度体系，加强行政监察和审计监督。认真落实全面从严治党要求，深入推进反腐倡廉建设，严肃查处各类腐败案件。

二要推进依法行政。推动政府部门和工作人员尊法学法守法用法，把政府工作全面纳入法治轨道。严格落实“三张清单”，完善重大行政决策程序，加强政府立法和行政执法监督、行政复议、行政应诉工作。认真执行市人大及其常委会决定决议，做好人大代表建议和政协提案办理工作，自觉接受各方面监督。深入推进政务公开，及时回应社会关切。

三要谋大事抓落实。政府全体工作人员要强化学习意识，积极适应经济新常态，刻苦学习新知识，加强调查研究，不断增强干事创业的能力和本领。强化创新意识，树立世界眼光和战略思维，科学谋划事关长远的大思路、大政策、大项目，努力向上争取支持。强化争先意识，坚持问题导向，提升工作标杆，积极争先进位。强化担当意识，以“钉钉子”精神抓好工作落实，只为成功想办法，不为落后找理由。健全激励机制和容错纠错机制，为改革创新者撑腰鼓劲，为敢于担当者担当。

四要提高行政效能。深入推进简政放权、放管结合、优化服务，创新事中事后监管方式。大力推行“互联网+政务服务”，加强政务服务平台和“中介超市”建设，提高工作效率，提升服务水平。建立完善惩戒机制，整治“庸懒散”，破解“中梗阻”，力戒不作为、慢作为。

各位代表！使命重在担当，实干铸就辉煌。让我们更加紧密地团结在以习近平同志为核心的党中央周围，在省委、省政府和市委的坚强领导下，紧紧依靠全市人民，凝心聚力，真抓实干，践行新理念，推动新发展，为率先全面建成小康社会、加快建设美丽幸福新东营而努力奋斗！
